# Domestic violence calls for police service in five US cities during the COVID-19 pandemic of 2020

**DOI:** 10.1186/s12889-022-14901-3

**Published:** 2022-12-29

**Authors:** Tesleem Babalola, Tianna Couch, Morgan Donahoe, Rachel Kidman, Amy Hammock, Rebecca Monastero, Douglas Hanes, Jaymie Meliker

**Affiliations:** 1 Program in Public Health, Stony Brook University, Stony Brook, NY USA; 2 Pomona College, Claremont, CA USA; 3 Westhampton Beach Senior High School, Westhampton Beach, NY USA; 4 School of Social Welfare, Stony Brook University, Stony Brook, NY USA; 5 School of Medicine, Stony Brook University, Stony Brook, NY USA; 6 Centre for Gender and Sexual Minority Health, Dalla Lana School of Public Health, University of Toronto, Toronto ON, Canada

**Keywords:** Domestic violence, COVID-19, Pandemics, Coronavirus, Intimate Partner violence, Domestic abuse

## Abstract

**Background:**

When COVID-19 stay-at-home orders were instituted, there were concerns that isolation may lead to increases in domestic violence (DV). Reports of increased rates of DV during the stay-at-home period have been suggestive of this but inconsistent across different locations. We sought to complement the existing studies by characterizing changes in DV trends in US cities of Chicago, Los Angeles (LA), New York City (NYC), Philadelphia, and Phoenix using police call volume data from January 1st, 2018, through Dec 31st, 2020.

**Methods:**

The stay-at-home orders were generally instituted for most US states in the second half of March 2020. We used the call volume for the pre-COVID-19 period (Jan. 2018 to Feb. 2020) to model a forecast against the stay-at-home order period (Mar. - May 2020) and the period after lifting the order (June – Dec. 2020) using the interrupted autoregressive integrated moving average (ARIMA) time series model.

**Results:**

During the stay-at-home order, increases in mean DV calls relative to pre-COVID-19 were observed in Chicago (47.8%), Phoenix (18.4%), NYC (3.5%), and LA (3.4%), but a decrease in Philadelphia (-4.9%). After lifting the stay-at-home order, changes in mean calls relative to pre-COVID-19 remained elevated in Chicago, slightly elevated in Phoenix, and returned to baseline in NYC and LA.

**Conclusion:**

Results suggest that the stay-at-home orders may have contributed to an increase in DV calls in some cities (Phoenix, and to a smaller extent LA, NYC), but the increase seen in Chicago (and to some extent Phoenix) persisted beyond the stay-at-home order and therefore may not be attributable to the stay-at-home orders. Additional studies are needed to help explain why the association between stay-at-home orders and DV police call volume seems to only appear in some locations.

## Introduction

Domestic violence (DV) generally refers to various types of violence (emotional, psychological, physical, and sexual) in the family or household, such as intimate partner violence (IPV), child abuse, and violence against any household member [1]. DV is a widespread issue in the United States, affecting an estimated 10 million individuals annually, with one in four women and one in nine men victims [2]. In response to the spread of COVID-19 in 2020, countries announced stay-at-home orders and closed schools and businesses. Key risk factors for DV, including job loss, school closures, and business closures, were present with lockdown conditions [3]. Unemployment rates in April of 2020 spiked to the highest level recorded in the United States since the Great Depression of the 1930s [4]. An environment of civil and economic unrest has previously increased DV incidence. For example, unemployment and economic hardship following the Great Recession of 2008 to 2009 were positively correlated with abusive behavior [5]. There was an association between hardship and men’s violent and controlling behavior toward their wives and partners [5].

The stay-at-home orders also affected mental health (heightened risk of depression, anxiety, stress-related disorders, and anger), which may impact domestic violence [6]. A study of Australian women in a cohabiting relationship reported an association between the COVID-19 pandemic and increased risk of relationship violence likely resulting from a combination of economic stress and social isolation [7]. Social isolation through stay-at-home orders could likely increase the time victims spend with abusers of DV [8].

Not surprisingly then, some studies suggest the COVID-19 pandemic increased DV; however, results are not uniform across cities and countries. A systematic review of DV during the pandemic period showed eight studies with decreased rates of DV, and 29 studies with increased rates of DV, with an average increase of 6–7% compared with previous years [9]. For example, in one study across 14 cities in the US, DV police calls increased by 7.5% during March through May of 2020, with effects concentrated during the first five weeks after social distancing began [10]. Another study reported an average 5% increase in DV incidence across 35 US cities from March to May 2020 [11]. A survey making use of a list randomization experiment reported an 8.3% increase in DV in Peru [12]. A study in Atlanta, however, showed increased rates of DV crimes in 2020 but those increases were highest in January of 2020, before the pandemic began [13]. In Ottawa, Ontario, there were decreases in emergency department visits for domestic and sexual violence during the early stages of the pandemic [14].

Collectively there are suggestions of increased risk of domestic violence, but results are not consistent across different locations. We sought to complement the existing studies by characterizing changes in DV trends in US cities of Chicago, Los Angeles, New York, Philadelphia, and Phoenix using police call volume data.

## Methods

DV-related calls to the police make up the single largest category of calls for service, accounting for 15 to more than 50% of all calls [15]. We retrieved calls-for-service data from five major cities in the United States with publicly available DV call police data: Los Angeles (LA), CA, Chicago, IL, New York City (NYC), NY, Philadelphia, PA, and Phoenix, AZ. The obtained data were publicly released as part of the Police Data Initiative via the Freedom of Information Act (FOIA). Our analysis includes monthly DV calls-for-service from January 2018 through Dec 2020 in all five cities. Calls-for-service included in the monthly totals were those coded as DV-related by each city. The annual estimated residential population of the cities from 2018 to 2020 was retrieved from United States census bureau data to calculate the rate of calls per 1000 residents for each year. Confidence interval (95%CI) was estimated for changes and percentage changes in calls based on stay-at-home order for each city using the CI for a difference between means [16] and exact binomial CI for proportion [17].

This is an ecological time series study using the call volume for the pre-COVID-19 period (Jan. 2018 to Feb. 2020) to model a forecast against the stay-at-home order period (Mar. - May 2020) and the period after lifting the order (June – Dec 2020) using the interrupted autoregressive integrated moving average (ARIMA) time series model (using SPSS; version 28.0). We specified a seasonality trend, and an autoregressive component dependent on the previous month’s value. This analysis was intended to quantify and visualize changes in volume of calls-for-service coded as DV in relation to stay-at-home orders and subsequent events during the COVID-19 pandemic within each city. We limited this to a within-city analysis, as opposed to a between-city analysis because of unidentified confounding factors such as social, demographic, and economic differences between cities.

## Results

In 2018 and 2019, New York City had the highest absolute mean monthly number of DV calls received by police (more than 15,500), while Chicago had the highest number of calls adjusted for the population (est. 4.6 calls per 1000 residents). In 2020, there was a relative increase in the rate of DV-related calls in Chicago (2.0 more calls/1000 residents) and Phoenix (0.2 more calls/1000 residents) but no significant increase in the other cities (Table 1).

**Fig. 1:**
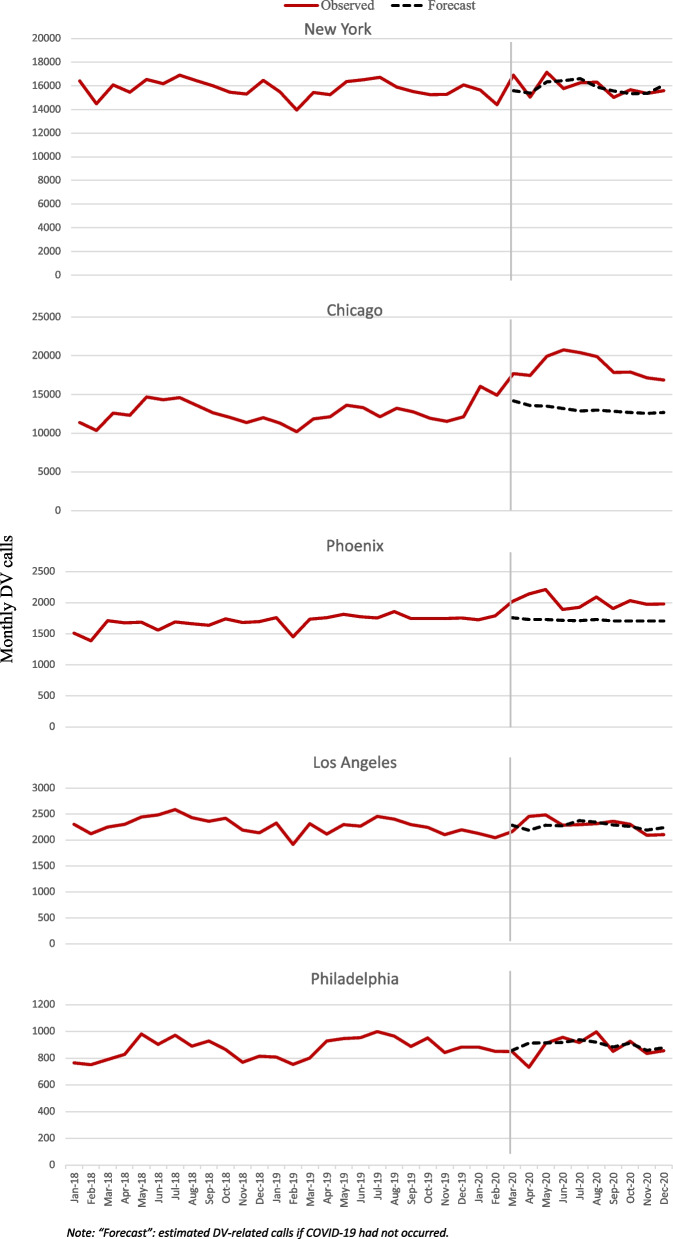
Monthly DV calls for each city: observed data and time series forecast.

The stay-at-home orders were generally instituted for most states in the country between March and May 2020. Therefore, event periods for the comparison with the forecast from the interrupted time-series analysis were specified at March and June 2020 for the stay-at-home order and lifting of order, respectively. During the stay-at-home order, increases in mean monthly DV calls relative to pre-COVID-19 were observed in Chicago (47.8%), Phoenix (18.4%), NYC (3.5%), and LA (3.4%), but a decrease in Philadelphia (-4.9%) (Table 2; Fig. 1). Differences were statistically significant in Chicago and Phoenix. After lifting the stay-at-home order, changes in mean calls relative to pre-COVID-19 increased by another 2.6% in Chicago to 50.4%, suggesting that the increase in Chicago may not be attributable to the stay-at-home order. Of note in Fig. 1, Chicago also saw increases in DV calls early in 2020 prior to the pandemic, also suggesting that increases in Chicago may not be attributed to COVID-19 or stay-at-home orders. However, in Phoenix, NYC, and LA, the number of DV calls moved closer to pre-COVID-19 levels, suggesting a possible impact of the stay-at-home order in those cities, followed be a return toward baseline after the stay-at-home order ended (Table 2).

**Table 1:** Descriptive analysis of monthly DV calls in each city: 2018–2020

Cities	2018			2019			2020		
	Mean (SD) monthly calls	Rate of calls per 1000 residents	95% CI	Mean (SD) monthly calls	Rate of calls per 1000 residents	95% CI	Mean (SD) monthly calls	Rate of calls per 1000 residents	95% CI
New York	15,981 (679)	1.90	(1.85, 1.95)	15,649 (741)	1.89	(1.84, 1.96)	15,760 (788)	1.79	(1.74, 1.86)
Chicago	12,667 (1382)	4.71	(4.37, 5.03)	12,182 (955)	4.51	(4.28, 4.72)	18,079 (1822)	6.62	(6.18, 7.02)
Phoenix	1637 (102)	1.00	(0.96, 1.04)	1741 (98)	1.01	(0.96, 1.04)	1974 (139)	1.18	(1.15, 1.25)
Los Angeles	2338 (144)	0.59	(0.58, 0.62)	2245 (146)	0.59	(0.58, 0.62)	2253 (145)	0.59	(0.58, 0.62)
Philadelphia	855 (80)	0.50	(0.47, 0.53)	893 (77)	0.60	(0.58, 0.63)	880 (68)	0.58	(0.57, 0.63)

Note: Annual estimated population retrieved from the United States census bureau (https://www.census.gov/ ) [18]

**Table 2:** Comparative analysis of DV calls pre-COVID-19, during stay-at-home order, and lifting of order

Cities	Mean Monthly DV calls		Changes in calls	95% CI of changes in calls	%Changes in calls	95% CI of % changes in calls
	Pre-COVID-19 (Jan 2018 – Feb 2020) [P]	COVID-19 [L]	(Cc) [C_**C**_ = L-P]		[%Cc =(C_**C**_ /P) *100]	
	**Stay-at-home order** (Mar 2020)					
New York	15,815	16,365	550	(-403, 1503)	3.48	(3.20, 3.77)
Chicago	12,425	18,368	5943	(4151, 7735)	47.83	(46.95, 48.71)
Phoenix	1793	2123	330	(191, 469)	18.40	(16.64, 20.28)
Los Angeles	2292	2369	77	(-114, 268)	3.36	(2.66, 4.18)
Philadelphia	874	831	-43	(-141, 55)	-4.92	(-3.58, -6.57)
	**Stay-at-home order lifted** (Jun 2020)					
New York	15,815	15,709	-106	(-842, 630)	-0.67	(-0.55, -0.81)
Chicago	12,425	18,693	6268	(4775, 7761)	50.45	(49.56, 51.33)
Phoenix	1793	1972	179	(72, 287)	9.98	(8.63, 11.46)
Los Angeles	2292	2252	-40	(-196, 116)	-1.75	(-1.25, -2.37)
Philadelphia	874	906	32	(-45, 109)	3.66	(2.52, 5.13)

*Cc=*Changes in calls relative to pre-COVID-19, *%Cc =*Percentage change relative to pre-COVID-19

## Discussion

Stay-at-home orders and economic downturns can affect communities and have devastating impacts beyond the grief caused by the pandemic. DV is one such possible unintended cost. In comparison with the pre-COVID-19 period, there were increases in DV police calls in Chicago, Phoenix, NYC, and LA, but not in Philadelphia during the stay-at-home period. Results were statistically significant in Chicago and Phoenix. However, after accounting for increases in calls in the period after the stay-at-home order was lifted, we see a net increase of within 3–8% in DV calls during the stay-at-home period in NYC, LA, and Phoenix; a decrease in DV calls in Philadelphia, and no net increase in DV calls in Chicago just during the stay-at-home period. We interpret our results as partially consistent with other studies on COVID-19 stay-at-home orders and DV which report an increase in DV by 5–7% [9, 11]. We unfortunately are unable to explain why the changes in DV call volume are not consistent across cities; these inconsistencies across locations are in line with previous studies [9].

Our study has several limitations that limit our interpretation of findings. The nature of DV can make reporting difficult. It is estimated that domestic abuse is reported only 2.5–15% of the time [19]. There are many reasons that reporting DV can be difficult; for example, some victims may experience financial barriers, intimidation, or isolation that prevent them from reporting their abuse. As a result of the pandemic and stay-at-home orders, more neighbors and community members were at home, and they may have heard instances of DV or become more aware of DV in their communities as they were spending more time at home. For example, in one study, increases in DV calls for service were more dramatic in areas with high population density [20]. The nature of reporting will be dependent on the victims’ environment and circumstances during the pandemic [21] and therefore interpretation of changes in DV calls over time may reflect social circumstances beyond only an increase in DV.

We therefore cannot directly link factors such as economic downturns or isolation as a cause for the increase in calls for service in this study. However, previous studies have suggested a link between increased DV, economic stress, and social isolation [11, 22, 23]. A survey conducted in Canada reported that inability to meet financial obligations and maintain social ties significantly increases family stress and DV [21]. Of note, an increase in reported DV incidents during sheltering in place order during the COVID-19 pandemic in some US cities has been reported to be driven by households without prior history of DV [10].

The source of our data on DV, police calls for report, merits discussion. In some instances, DV calls-for-service are reported by third-party members, some calls do not warrant police response, and some calls may be false reports. Finally, the severity of situations warranting calls is not captured in the call for services data. Not all DV calls are substantiated, and not all DV is reported; thus, a limitation is the use of call for services as the primary data source. In addition, we used census data to estimate the underlying population when calculating rates of DV reports, yet we know that the population shifted during the pandemic, up to 10% in some cities, with many wealthier individuals relocating out of urban areas. However, we do not know the extent to which those who relocated were experiencing domestic violence so the impact of this population shift on our study is not simple to discern. Despite these limitations, these findings suggest that along with significant rates of psychological distress [24] as well as increasing reports of substance withdrawal emergencies and death [25], DV perpetration and victimization were also impacted by the social isolation brought about by the pandemic, although unevenly across cities.

## Conclusion

It is imperative that public health practitioners and policymakers develop protocols to better reach out to isolated vulnerable individuals, especially during times of social isolation. Capturing intimate partner violence and family violence data is essential to support men, women, children, and families affected by violence. DV-related data could provide information on which interventions can be adopted to reduce future DV incidence. Financial hardships and stress coupled with school closures and stay-at-home orders can have drastic effects on families and childhood development. Public health officials, as well as policymakers and police services, need to consider intervention strategies and resources that can be utilized in unforeseen adverse periods such as pandemics.

## Data Availability

Datasets used for this study are publicly available DV call police data. We contacted each police department in Chicago, Los Angeles, New York City, Philadelphia, and Phoenix to request and gain access to their data; in some cases, freedom of information act paperwork was required and was submitted. *Chicago*: https://home.chicagopolice.org/statistics-data/data-requests/. *Los Angeles*: https://www.lapdonline.org/statistical-data/. *New York City*: https://www.nyc.gov/site/nypd/stats/crime-statistics/crime-statistics-landing.page. *Philadelphia*: https://www.phillypolice.com/crime-maps-stats/. *Phoenix*: https://www.phoenixopendata.com/organization/police-department.
